# Supplementary material for: Natural and synthetic inhibitors of a phage-encoded quorum-sensing receptor affect phage–host dynamics in mixed bacterial communities
Source: Proc Natl Acad Sci U S A. 2022 Nov 29;119(49):e2217813119. doi: 10.1073/pnas.2217813119 (PMC9894119; doi:10.1073/pnas.2217813119)
Supplement: Supplementary file 1 — Appendix 01 (PDF) [file pnas.2217813119.sapp.pdf]

Supporting Information for

Natural and synthetic inhibitors of a phage-encoded quorum-sensing receptor affect phage-host dynamics in mixed bacterial communities

Justin E. Silpe[1,2,3], Olivia P. Duddy[1,3], and Bonnie L. Bassler[1,2,*]

1 Department of Molecular Biology, Princeton University, Princeton, NJ, USA

2 Howard Hughes Medical Institute, Chevy Chase, MD, USA

3 These authors contributed equally

Contents: Item | Description

Supplementary Figure S1. The ARM81 *ahyI* gene encodes the C4-HSL-producing synthase AhyI and both C4-HSL and C8-HSL solubilize $LuxR_{ARM81ld}$.

Supplementary Table S1. Strains used in this study.

Supplementary Table S2. Primers used in this study.

Supplementary Table S3. Plasmids used in this study.

Figure S1

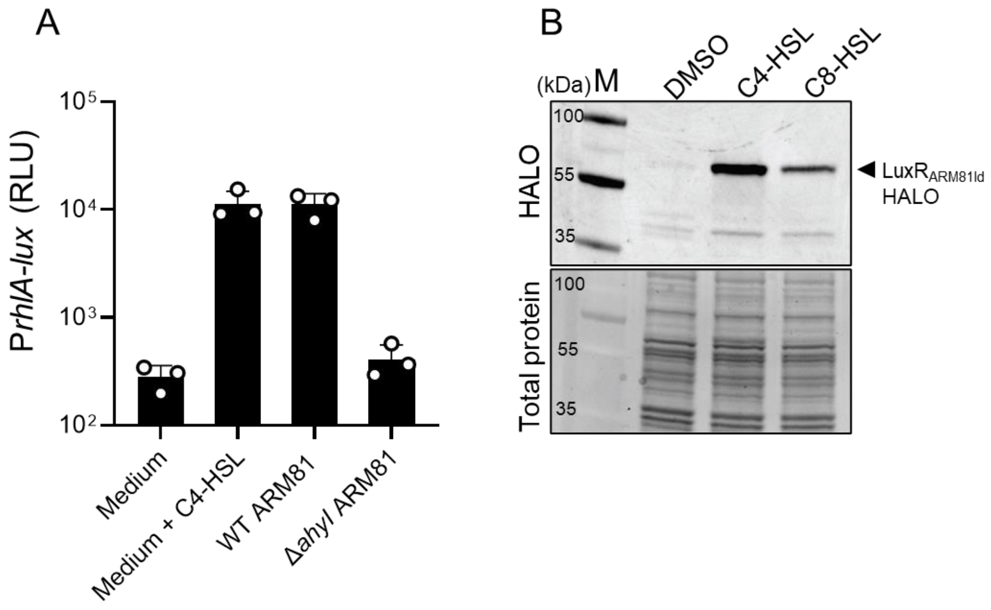

Figure S1. The ARM81 *ahyI* gene encodes the C4-HSL-producing synthase AhyI and both C4-HSL and C8-HSL solubilize LuxR$_{ARM81ld}$.

(A) P*rhlA*-*lux* expression from *E. coli* grown in medium supplemented with C4-HSL, cell-free culture fluids from WT *Aeromonas* sp. ARM81 (C4-HSL$^+$), or cell-free culture fluids from Δ*ahyI Aeromonas* sp. ARM81 (C4-HSL$^-$). The *E. coli* strain carries a plasmid with P*rhlA-lux* and a second plasmid with arabinose-inducible pBAD-*rhlR*. The *rhlA* promoter and *rhlR* gene both come from *P. aeruginosa.* In this system, RhlR only activates P*rhlA*-*lux* expression when C4-HSL is supplied exogenously. All media contained 0.1% arabinose.

(B) Western blot (top) and total protein (bottom) showing LuxR$_{ARM81ld}$-HALO in the soluble fractions of *E. coli* supplied with 75 µM of the indicated HSL or an equivalent volume of DMSO. Detection of the HALO-reactive fluorophore requires that the HALO tag be properly folded. Thus, LuxR$_{ARM81ld}$-HALO band intensity is a measure of folded LuxR$_{ARM81ld}$ in the soluble fraction of each sample. M denotes molecular weight marker (representative bands are labeled). kDa is kilodalton.

Table S1. Strains used in this study.

Strain	Genotype	Reference
E. coli TOP10	F– *mcrA* Δ*(mrr-hsdRMS-mcrBC)*<br>*φ80lacZ*Δ*M15* Δ*lacX74 recA1 araD139*<br>Δ*(ara-leu)7697 galU galK*<br>*λ– rpsL*(Str^{R}) *endA1 nupG*	Invitrogen
E. coli BL21	*fhuA2 [lon] ompT gal [dcm]* Δ*hsdS*	Invitrogen
Aeromonas sp. ARM81 (JSS-3155)	WT	(Dziewit and Radlinska, 2016)
Aeromonas sp. ARM81 (JSS-3388)	Δ*ahyI*	This study
Vibrio fischeri ES114	WT	Bassler lab strain collection
	Δ*luxI*	Bassler lab strain collection
	Δ*ainS*	Bassler lab strain collection
	Δ*luxI* Δ*ainS*	Bassler lab strain collection

Table S2. Primers used in this study.

Name	Sequence (5 - 3)	Purpose; Template
JSO-2192	GTTTGCGGCCGCACGTTTTTACCCCC CCAAAGCC	*ahyI* upstream flanking DNA for insert into pFOG with NotI site; from *Aeromonas* sp. ARM81
JSO-2193	TTAACACTCTGTTATTACAAATGAGCA AAAAAAGTCCCGGGA	*ahyI* upstream flanking DNA for insert into pFOG; from *Aeromonas* sp. ARM81
JSO-2194	TTTGTAATAACAGAGTGTTAATT	*dhfr* cassette amplification; from EZ-DHFR Tn*5*
JSO-2195	TTAATTAAGCCTGGCACAGCGG	*dhfr* cassette amplification; from EZ-DHFR Tn*5*
JSO-2196	CGCTGTGCCAGGCTTAATTAACATTC ATGCCTCTCCATTTCA	*ahyI* downstream flanking DNA for insert into pFOG; from *Aeromonas* sp. ARM81
JSO-2197	GTTTTGTTTAAACACATGGGGATGCG TTCCTGTGA	*ahyI* downstream flanking DNA for insert into pFOG with PmeI site; from *Aeromonas* sp. ARM81
JSO-1566	CATTAATTAACCTCCTGAATTCGGAA GCGAT	pT7 backbone amplification for Gibson assembly; pH6HTN plasmid
JSO-2035	TAATAGTCAACCGCTGAGCAATAACT AGC	pT7 backbone amplification for Gibson assembly; pH6HTN plasmid
JSO-2176	GCTTTGTATATGCCCCCACCTA	qPCR; phage ARM81ld
JSO-2177	GCAATCAGACATCAGTGCCAAG	qPCR; phage ARM81ld
JSO-2106	CTTCTCTTCCGAGCGTTACGAC	qPCR; *Aeromonas* sp. ARM81 host (*rpoB*)
JSO-2107	GGTCGATATCGTCCACTTCGTC	qPCR; *Aeromonas* sp. ARM81 host (*rpoB*)

Table S3. Plasmids used in this study.

Plasmid name (informal)	Strain ID (formal)	Relevant fragment	Marker, Origin	Source
pTetA-$xre_{ARM81ld}$-$luxR_{ARM81ld}$	JSS-3237	$xre_{ARM81ld}$-$luxR_{ARM81ld}$	Amp, pBR322	(Silpe *et al.,* 2022)
pTetA-$xre_{ARM81ld}$-$luxR_{ARM81ld}$	JSS-3326	$xre_{ARM81ld}$-$luxR_{ARM81ld}$	Cm, p15A	(Silpe *et al.,* 2022)
P$smORF_{ARM81ld}$-*lux*	JSS-3235	P$smORF_{ARM81ld}$-*lux*	Kan, p15A	(Silpe *et al.,* 2022)
pFOG-Δ*ahyI::dhfr*	JSS-3387	Δ*ahyI::dhfr*	Gent, oriR6kγ	This study
pH6HTC-pT7-$luxR_{ARM81ld}$-*HALO-HIS*	JSS-1868	$luxR_{ARM81ld}$-*HALO-HIS*	Amp, pBR322	(Silpe and Bassler, 2019)
